# Extraperitoneal Robot-Assisted Radical Prostatectomy with the Hugo™ RAS System: Initial Experience at a High-Volume Robotic Centre

**DOI:** 10.3390/jcm13195916

**Published:** 2024-10-03

**Authors:** Marcello Scarcia, Giovanni Battista Filomena, Stefano Moretto, Filippo Marino, Simone Cotrufo, Alessandra Francocci, Francesco Paolo Maselli, Giuseppe Cardo, Giovanni Pagliarulo, Pierluigi Rizzo, Pierluigi Russo, Michele Di Dio, Stefano Alba, Roberto Calbi, Michele Romano, Michele Zazzara, Giuseppe Mario Ludovico

**Affiliations:** 1 Department of Urology, “F. Miulli” General Hospital, 70021 Acquaviva delle Fonti, BA, Italy; scarciam@hotmail.com (M.S.); stefano.moretto.ch@gmail.com (S.M.); s.cotrufo@miulli.it (S.C.); alessandra.francocci@gmail.com (A.F.); f.maselli@miulli.it (F.P.M.); g.cardo@miulli.it (G.C.); g.pagliarulo@miulli.it (G.P.); p.rizzo@miulli.it (P.R.); m.romano@miulli.it (M.R.); michele.zazzara@gmail.com (M.Z.); giuseppeludovico@hotmail.com (G.M.L.); 2 Department of Urology, Fondazione Policlinico Universitario A. Gemelli IRCCS, Università Cattolica del Sacro Cuore, 00168 Rome, Italy; pierluigi92.russo@gmail.com; 3 Department of Urology, Humanitas Clinical and Research Institute IRCCS, Rozzano, 20089 Milan, Italy; 4 Department of Biomedical Sciences, Humanitas University, 20072 Milan, Italy; 5 Division of Urology, Department of Surgery, SS Annunziata Hospital, 87100 Cosenza, Italy; micheledidio@yahoo.it; 6 Department of Urology, Romolo Hospital, 88821 Rocca di Neto, KR, Italy; stefanoalba78@gmail.com; 7 Department of Radiology, “F. Miulli” General Hospital, 70021 Acquaviva delle Fonti, BA, Italy; r.calbi@miulli.it

**Keywords:** prostate cancer, robotic surgery, Medtronic Hugo RAS system, robot-assisted radical prostatectomy, new robotic platform, surgical outcomes

## Abstract

**Background:** The Hugo™ Robotic-Assisted Surgery (Hugo™ RAS) system represents a novel advancement in robotic surgical technology. Despite this, there remains a scarcity of data regarding extraperitoneal robot-assisted radical prostatectomy (eRARP) using this system. **Methods:** We conducted a prospective study at Ospedale Regionale “F. Miulli” from June 2023 to January 2024, enrolling consecutive patients diagnosed with prostate cancer (PCa) undergoing eRARP ± lymph node dissection. All procedures employed a modular four-arm setup performed by two young surgeons with limited prior robotic surgery experience. This study aims to evaluate the safety and feasibility of eRARP using the Hugo™ RAS system, reporting comprehensive preoperative, intraoperative, and postoperative outcomes in the largest reported cohort to date. **Results:** A total of 50 cases were analyzed, with a mean patient age of 65.76 (±5.57) years. The median operative time was 275 min (Q1–Q3 150–345), and the console time was 240 min (Q1–Q3 150–300). The docking time averaged 10 min (Q1–Q3 6–20). There were no intraoperative complications recorded. Two major complications occurred within the first 90 days. At the 3-month mark, 36 patients (72%) achieved undetectable PSA levels (<0.1 ng/mL). Social continence was achieved by 66% of patients, while 40% maintained erectile function. **Conclusions:** eRARP utilizing the Hugo™ RAS system demonstrated effectiveness and safety in our study cohort. However, more extensive studies with larger cohorts and longer follow-up periods are necessary to thoroughly evaluate long-term outcomes.

## 1. Introduction

The most common cause of cancer-related deaths in Western countries is prostate cancer (PCa), which primarily affects men in their middle years, usually between the ages of 45 and 60. The precise genetic mutation pattern underlying PCa is still not fully understood [1,2]. Over the past two decades, robot-assisted surgical systems have seen substantial advancements, with several new robotic platforms now emerging as advanced solutions and strong competitors for market leadership [3]. Robotic surgery is currently a major component of urology; over 90% of radical prostatectomies in the U.S. are now performed using robotic systems, with similar growth seen in Europe. The magnified image simplifies this integration by helping to identify anatomical differences and optimize cancer control while maintaining erection and continence [4,5]. For men with localized prostate cancer, radical prostatectomy is still the preferred method of treatment. Globally, robot-assisted surgery is becoming increasingly widespread across several surgical specialties. Although this approach has shown lower rates of readmission and blood loss, it has not demonstrated significant superiority in functional or oncological outcomes compared to the open approach [6,7]. Furthermore, controversial and debated research has indicated that robotic radical prostatectomy (RARP) may potentially improve postoperative erectile function and achieve better rates of urinary continence compared to alternative approaches [8].

The da Vinci platform from Intuitive Surgical Inc. has dominated robotic surgery for the last 20 years [9]. With Intuitive’s expired patent, numerous competing technologies are entering the market [10]. The emergence of these innovative robotic platforms presents an opportunity to reduce costs and expand the accessibility of robotic surgery. The Hugo™ Robotic-Assisted Surgery (RAS) system stands out as a potential new platform with unique features, including an open console, a system tower, and independent arm carts. Its portability and versatility are among its most distinctive features. Additionally, the safety and reliability of radical prostatectomy with a transperitoneal approach have been confirmed in several studies [11,12,13,14,15]. However, limited data on using extraperitoneal RARP (eRARP) with the Hugo™ RAS system are available. Therefore, this research aims to investigate safety and viability and report outcomes in the largest cohort undergoing eRARP with the Hugo™ RAS system (Hugo™ RAS).

## 2. Materials and Methods

We designed a prospective clinical study to evaluate the performance of the Hugo™ RAS system (Medtronic, Minneapolis, MN, USA) at our center, Ospedale Generale Regionale F. Miulli, Acquaviva delle Fonti, Italy, and to examine the surgical and perioperative outcomes of patients undergoing eRARP during an initial short-term follow-up period. The study strictly adhered to the Declaration of Helsinki and Good Clinical Practice principles. Prior to initiation, ethical approval was secured from the institutional ethics committee (No. 6331), and informed consent was obtained from all patients participating before their surgical procedures. All patients provided consent for the collection of intraoperative and postoperative data, ensuring adherence to standard clinical practices and guidelines without any deviations. Additionally, our center did not identify any specific rationale for selecting the Hugo™ robotic platform over the long-established da Vinci robotic system. The surgeons and operating room nurses involved in the study all received technical training at the ORSI Academy in Aalst, Belgium. Fifty consecutive patients with biopsy-proven, pelvic-confined PCa who needed therapeutic surgery were included in the study. Between June 2023 and January 2024, they underwent eRARP ± pelvic lymph node dissection (PLND) with the Hugo™ RAS system.

The primary goal of the study was to record the Hugo RAS system’s surgical approach and assess its feasibility for performing eRARP. Secondary objectives included reporting intraoperative and short-term associated outcomes. Except in cases where it was not appropriate (such as end-stage renal disease), every patient evaluated for RARP had a multiparametric magnetic resonance imaging (MRI) before a prostate biopsy. Data were collected preoperatively, intraoperatively, and postoperatively. Postoperative complications were categorized using the Clavien–Dindo classification system. Data collection was managed using a prospective Microsoft Excel database (2311 build 17029.20108). Continuous variables were expressed as means with standard deviations (SDs), whereas non-normally distributed data were presented as medians along with the interquartile range (Q1–Q3). The prevalence data were presented as a percentage of all observations.

Preoperative staging was conducted based on the EAU risk classification for biochemical recurrence of localized or locally advanced PCa. Patients categorized as high or very high risk, as well as those in the highest subgroup of intermediate risk, underwent the necessary investigations. For staging, either PET-CT with choline or a whole-body CT scan combined with bone scintigraphy were used. Exclusion criteria encompassed instances of incomplete data and refusal to provide informed consent. All eRARP procedures were performed using a modular four-arm configuration by two young surgeons with limited prior experience in robotic surgery. Each surgeon had only completed ten previous procedures, all utilizing the HUGO RAS system. Pelvic lymph node dissection (PLND) was undertaken in patients whose preoperative assessment indicated a nodal involvement risk exceeding 5% [16] or 7% [17]. Nerve-sparing procedures were performed based on the risk of ipsilateral extracapsular extension, determined by factors such as clinical T stage, ISUP grade, magnetic resonance imaging findings, and consultation of Partin tables. We collected all perioperative data prospectively, and an independent researcher gathered postoperative data up to April 2024 through follow-up visits or scheduled telephone interviews at 1 and 3 months post-surgery. We also reported data on the social continence rate, defined as the need for up to one pad daily [18]; unfavorable positive surgical margins (PSMs), characterized by either a single positive margin of ≥3 mm or multifocal positive margins [19]; and erectile potency, assessed three months post-surgery, as the capability to get an erection sufficient for sexual intercourse, with or without the use of drugs (phosphodiesterase inhibitors).

Urinary catheters are typically removed from all patients within 10 to 15 days, following our internal protocol, contingent upon completing a cystography to confirm the absence of urinary extravasation. Additionally, patients are encouraged to engage in daily pelvic floor muscle exercises for three months following surgery.

### 2.1. Patient and Trocar Placement

**Figure 1:**
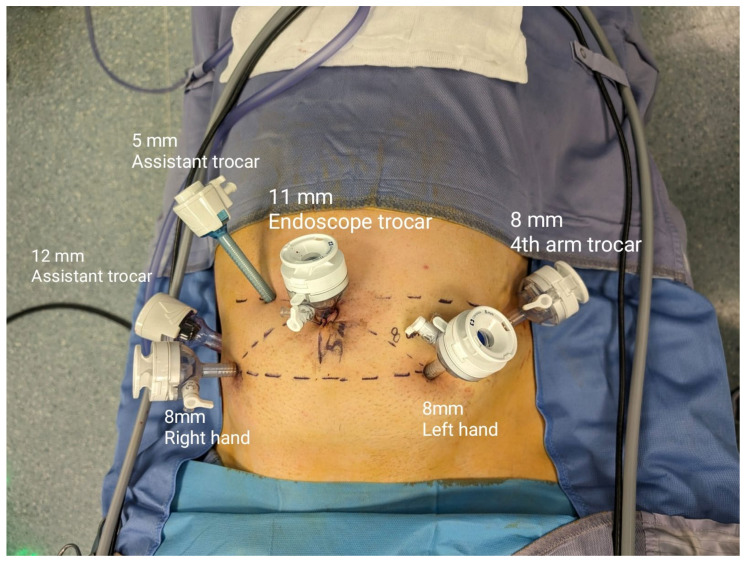
Trocar placement in extraperitoneal RARP (eRARP).

**Figure 2:**
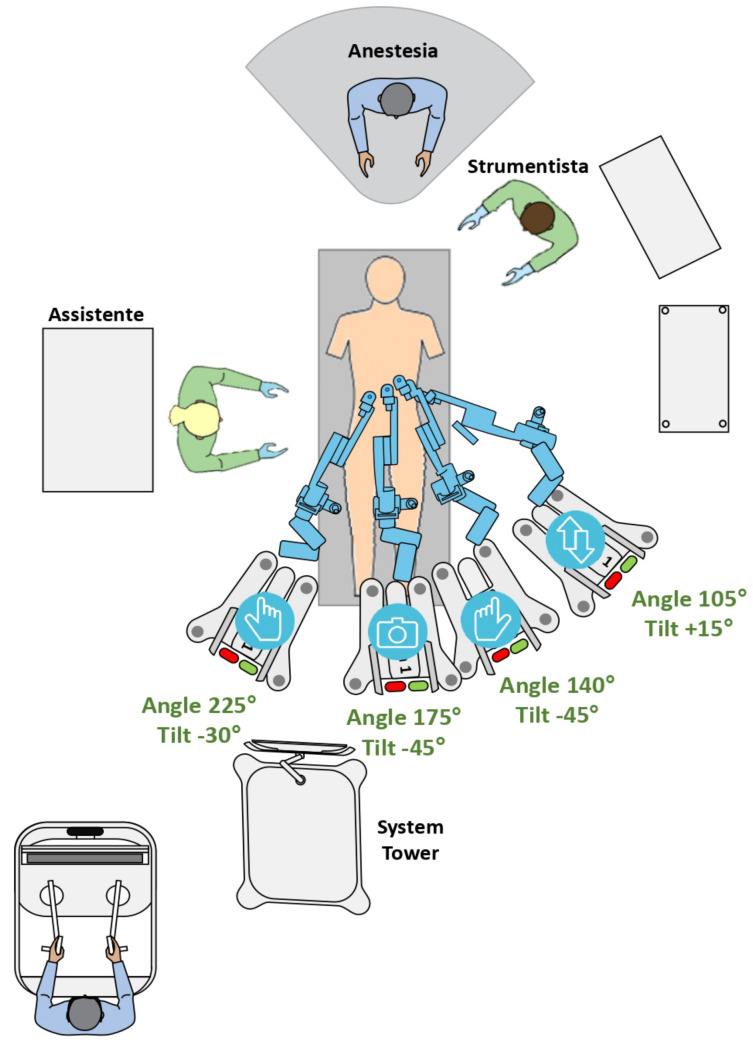
HUGO™ RAS system operative room settings, tilt and docking angles (DAs).

For the extraperitoneal approach, patients were placed in a low lithotomy position with a Trendelenburg tilt (20°), and the legs were abducted at 30°. We made a 1.5 cm midline longitudinal skin incision below the umbilicus, followed by an anterior rectus sheath incision. Using blunt finger dissection, the rectus abdominis muscle was moved laterally to expose the extraperitoneal space behind the posterior rectus sheath. After creating an adequate workspace, two 8 mm robotic trocars were placed 8 cm away from the camera port. On the right side, at the level of the umbilicus, a 5 mm assistant port was positioned. After achieving pneumoperitoneum under a 0° optical view, the W-shaped trocar positioning for RARP was followed by placing a 12 mm assistant port and an 8 mm third robotic port (Figure 1 and Figure 2).

### 2.2. Docking

To gradually decrease docking time, we implemented a systematic docking scheme. Two arm carts were positioned on the left side of the patient’s bed: one on the right, and one in the center as part of our setup. The energy tower remained positioned at the foot of the bed, with the scrub nurse on the patient’s left and the assistant on the right. The endoscope arm cart was first docked, approaching from between the patient’s spread legs. After that, the surgeon’s right-arm cart was docked from the right side of the bed, after docking the adjacent left-arm cart. The docking and tilting angles in our configuration were configured as follows: endoscope at 175°, −45°; surgeon’s left hand at 140°, −45°; surgeon’s right hand at 225°, −30°; and fourth arm at 105°, +15° (Figure 2).

### 2.3. Surgical Technique

The surgical procedure commenced with the removal of pre-prostatic fat to expose key anatomical landmarks necessary for performing a detrusor apron incision and bladder neck dissection. During this process, care was taken to preserve the integrity of the lateral endopelvic fascia, pubo-prostatic ligaments and deep venous complex. Following this, the bladder neck was dissected, the urethra was incised at the intra-prostatic level, and meticulous preservation of the vas deferens and seminal vesicle was ensured. Subsequently, the posterior stratum of Denonvillier’s fascia was incised, with the surgeon selecting the appropriate level of dissection, whether intrafascial or interfascial. Hem-o-lock clips were utilized to secure the prostatic pedicles during the anterograde dissection of the prostate, employing a blunt and athermal technique. Careful attention was given to avoid manipulation or electrocauterization, thereby minimizing potential damage to the neurovascular bundles separated from the prostate. The procedure aimed to preserve the maximum urethral length by isolating the prostatic apex and making an incision in the urethra below the verumontanum [20]. The surgical specimen was placed in the left lateral quadrant after being secured in an Endocatch bag to facilitate the subsequent anastomosis. Continuous 3-0 barbed sutures were employed to achieve complete posterior anatomical reconstruction. For the vesicourethral anastomosis, two semicontinuous 3-0 barbed sutures were positioned around a 20 Fr silicone catheter. All incisions were closed without sealing the fascial openings, except for the endoscope port, and a drain was inserted through the Retzius space. Nerve-sparing techniques were performed following the approach described by Tewari et al. [21].

## 3. Results

At our institution, RARP with an extraperitoneal approach was administered to fifty consecutively enrolled patients. The median body mass index was 27 kg/m^2^ (Q1–Q3 24–29), and the mean age was 65.76 (±5.57) years. Following a prostate biopsy, 16 patients (32%) were classified as grade group ≥ 3, according to the International Society of Urological Pathology (ISUP) recommendations. Table 1 provides an overview of all demographic and baseline data. None of the patients required intraoperative transfusions, with an estimated mean blood loss of 148 mL (±57.4). There were no intraoperative complications, and all planned procedural steps were completed without conversion or additional port placements. No notable intraoperative complications or technical issues impeding surgery completion were observed. The median total operating room time was 315 min (interquartile range [Q1–Q3] 220–405), with a median operative time of 275 min (Q1–Q3 150–345) and a median console time of 240 min (Q1–Q3 150–300). The median docking time was 10 min (Q1–Q3 9–10). The 35 min difference between console time and operative time, despite a 10 min docking time, is attributed to several factors. These include the lead surgeon assisting with trocar placement and docking, adjustments required for monitor positioning due to the small operating room, and the inclusion of post-console tasks in the operative time, such as drain placement, specimen removal, trocars removal under visualization and closing the incisions. Additionally, longer times during early cases contributed to the discrepancy. Twenty-six patients underwent standard pelvic lymph node dissection (PLND), and three patients were suspected of having pelvic lymph node involvement based on radiological findings (cN+). The median surgical time was approximately 32 min longer for cases involving lymphadenectomy, with times of 332 min (Q1–Q3 315–350), compared to 300 min (Q1–Q3 272.5–315) for cases without lymphadenectomy (see Table 2).

We used the standard PLND template for our procedures and were able to remove an average of nine lymph nodes per procedure (Q1–Q3 8–13). We reported data on the difference between preoperative and postoperative hemoglobin (Hb) levels, reported as the median, showing a postoperative decrease of 3.1 g/dL (Q1–Q3 2–4). To minimize complications such as delayed bowel function, nausea, and vomiting, our postoperative pain management protocol emphasizes reducing opioid use. Pain relief plans are customized for each patient, typically including up to 1 g of paracetamol three times a day, with NSAIDs and opioids administered as necessary. To ensure patient safety, any known allergies have been considered. Significantly, 66% of patients required paracetamol for pain management, whereas only 4% relied on opioids. Four days was the median length of hospital stay (Q1–Q3 3–5). Significant postoperative complications occurred in two patients during the first thirty days following surgery. A cystostomy was necessary for one patient to reposition the catheter, while an acute kidney injury due to hydronephrosis necessitated a bilateral percutaneous nephrostomy for another patient (Clavien–Dindo IIIa). Additionally, two patients underwent blood transfusions, one received anticoagulant therapy for a pulmonary embolism, and two more were treated with antibiotics for urinary tract infections (Clavien–Dindo II). No further complications were reported.

After pathology was completed, 20 patients (40%) had tumors classified as ISUP grade group 3–5, 13 (26%) had extraprostatic extension, and 3 (6%) had involvement of lymph nodes (see Table 3). The rate of positive surgical margins (PSMs) was 44% (22 patients), with 14 cases (63.6%) having margins greater than 3 mm. Within this cohort, one month following surgery, 80% of patients had an undetectable PSA (<0.1 ng/mL), and 72% were still at this level three months later. Regarding urinary continence (UC) recovery at 3 months, 36 patients (72%) achieved social continence [18,22]. Twelve patients (26%) required two or more pads per day, compared to the median of one pad (Q1–Q3 0–4) (see Table 3).

## 4. Discussion

In this single-center study utilizing the HUGO™ RAS system, we have demonstrated the implementation and feasibility of performing eRARP. For surgeons adapting to this technology, several features of this robotic platform are particularly noteworthy. The modularity of the robotic arms and the open console design, which features specialized hand controllers, represent significant innovations deserving careful consideration. The open console design allows for direct communication between the operating room staff and the robotic surgeon, enabling the use of external devices such as radiological images, ultrasound images and three-dimensional reconstructions. The surgeon utilizes specialized glasses to achieve high-resolution three-dimensional vision through a monitor, particularly beneficial in academic centers. Due to this modular design, the robotic arms are mounted on separate carts, establishing distinct modules that allow for various surgical configurations, including three or four-arm setups. However, compared to a single-cart platform, the four individual carts occupy more space and require additional storage. The arms and carts can be cumbersome, potentially restricting space for the second surgical operator. Various urological procedures have evaluated Hugo’s RAS system performance [23]. Early experiences with this robotic system indicate that transperitoneal RARP can achieve satisfactory perioperative and functional outcomes, with operative times comparable to those of DaVinci robotic systems [13,24]. Nevertheless, a limited number of eRARPs using the HUGO™ RAS system have already been published, with the focus being on tilt angles, arm positioning, trocar positioning, surgical feasibility and safety assessment [14,25].

Despite its low perioperative morbidity, the transperitoneal approach is still the most frequently performed RARP technique; however, it carries a risk of perforating the peritoneal cavity [26]. Considering its potential benefits over the transperitoneal approach, our institution has chosen to adopt the extraperitoneal approach for RARP procedures (eRARP). These advantages include the potential to utilize a reduced Trendelenburg position, decreased risk of intraperitoneal organ injury, lower incidence of complications from intraperitoneal urine leakage and enhanced surgical feasibility in obese patients or those with prior pelvic or abdominal surgery [27]. Nevertheless, the extraperitoneal approach has disadvantages, such as increased vesicourethral anastomosis tension, heightened lymphocele risk, and restricted operative space [28]. Studies with the Da Vinci robotic system have validated the safety and effectiveness of eRARP, demonstrating outcomes comparable to the transperitoneal approach [29]. We applied our in-depth knowledge of eRARP with the new HUGO™ RAS system in our study. Based on previously published research, we modified the trocar positioning scheme for our surgical techniques (see Figure 1 and Figure 2). This configuration was implemented during surgery to optimize the fourth arm’s maneuverability around all anatomical structures, particularly during the dissection of the left neurovascular bundle.

Recent reports have raised concerns about potential conflicts between scrubbed nurses and robotic arms. However, we securely completed all validated procedural steps without requiring conversion or additional adjustments. These findings suggest that our approach can be effectively implemented with the described setup. Because this study represents a case series, patient selection was not conducted. Among the patients, 20 (40%) had prior abdominal surgery, and 6 cases (12%) had undergone previous prostate adenomectomy. Additionally, 11 patients were classified as severely obese (BMI > 30, 22%) [30]. Notably, compared to the results of Bravi et al., our study’s median operative time and console time difference were more significant, which could be explained by our surgeons’ limited experience [31]. Nonetheless, a noteworthy obstacle to this robotic system is the docking procedure, which may increase surgical duration and pose difficulties. However, prior research has shown that a skilled team can shorten the initial docking time. Marino et al. conducted a systematic review and pooled analysis, reporting a median docking time of 11 min (95% CI 7.95–14.50 min) for transperitoneal RARP using the HUGO™ RAS system [15].

Although our extraperitoneal procedures employed a different docking technique, our results on docking time (median of 10 min) are consistent with the published literature. Our experience indicates that a proficient team, trained regularly and capable of simultaneously docking multiple carts, can significantly expedite the process following initial setup. Moreover, the acquisition of necessary skills was facilitated by the presence of a Medtronic specialist in the operating room, thereby accelerating the transition to independent docking and reducing overall setup times. Notably, our study’s estimated blood loss and hospital stay duration were comparable to those observed in high-volume robotic surgery centers, underscoring the approach’s positive performance with the robotic system [32]. An essential drawback of the extraperitoneal approach is its limitation in performing extensive PLND. There is still some disagreement about whether or not PLND is necessary for people with locally advanced prostate cancer. Because wider dissection templates come with higher risks without clear therapeutic benefits for oncological outcomes, we chose to use the standard PLND template in our procedures. Nevertheless, similar to transperitoneal RARP, recent studies also indicate the safety and oncological effectiveness of extended extraperitoneal PLND. Our series found that utilizing the Hugo™ RAS system to perform standard extraperitoneal PLND was straightforward, safe and completed within acceptable timeframes. This approach provided sufficient exposure regarding the necessary anatomical landmarks for conducting a typical PLND [33,34].

Furthermore, no intraoperative complications were observed in our study, which is consistent with earlier reports. We identified only two Clavien–Dindo IIIb complications, both requiring subsequent surgeries. One case necessitated percutaneous cystostomy placement, while the other involved bilateral percutaneous nephrostomy placement due to acute renal injury secondary to hydronephrosis. In both cases, the patients did not require further treatment after the cystostomy, and nephrostomies were removed 20 days after surgery. In total, 14% of patients had complications after surgery related to Clavien–Dindo ≥2. This is more than what was seen in other case series using the Hugo™ RAS system.

Regarding oncological outcomes, our study reported a notably higher incidence of positive surgical margins (44%), which surpasses both our previous experience with the da Vinci system [35] and the typical range reported in the literature (26% to 32%) [36,37]. This elevated rate of positive margins can be attributed to various factors, including patient-specific characteristics, the learning curve associated with the surgeon’s experience, and the inherent complexity of the procedure [38]. Furthermore, our data indicate a trend in which the incidence of positive surgical margins decreases with increasing surgeon experience. Specifically, we observed 15 positive margins among the first 25 patients compared to only 7 positive margins among the subsequent 25 patients. However, we also noted a minor drawback of the Hugo™ RAS system concerning traction phenomena: the lack of a dedicated robotic instrument for delicate grasping and traction tasks. This limitation was particularly evident in the posterolateral basal region, which undergoes substantial traction during the development of the posterior plane, alongside the isolation of the neurovascular pedicles, and was involved in 14 out of 22 (63.6%) surgical margins. Regarding oncological outcomes, 82% of patients in our case series achieved a PSA level lower than 0.1 ng/mL at one-month post-surgery. Among the twenty-two patients with positive surgical margins, four were classified as having very high-risk locally advanced disease based on the final histological assessment, and seven of these patients still had detectable PSA levels three months after surgery. In our sample of 50 patients, 72% had undetectable PSA at the 3-month post-surgery follow-up [39,40].

Regarding postoperative erectile function, erections were documented in 40% of patients at the 3-month post-surgery mark, consistent with findings reported in the existing literature [41]. We acknowledge several limitations, despite our study providing valuable insights into the feasibility and initial outcomes of extraperitoneal RARP using the Hugo™ RAS system. These include a small sample size, limited surgical experience, the absence of a control group, and reliance on descriptive data, which may restrict the generalizability of our findings. Furthermore, the short follow-up period limits the assessment of postoperative oncological outcomes and precludes direct comparisons with the existing literature.

For a global evaluation, a more extended follow-up period would be advantageous. Nevertheless, this study represents the initial investigation of surgical outcomes for extraperitoneal RARP using the Hugo™ RAS system. While we await further research with longer follow-up periods, our findings provide valuable data on perioperative, early oncological and functional outcomes for RARP performed using this innovative robotic platform.

A more comprehensive evaluation would benefit from a longer follow-up period. Nevertheless, this study marks the initial investigation of surgical outcomes for extraperitoneal RARP using the Hugo™ RAS system. Our findings offer valuable insights into perioperative, early oncological and functional outcomes associated with RARP performed using this innovative robotic platform, underscoring the need for further research with extended follow-up periods.

## 5. Conclusions

To the best of our knowledge, the presented case series is the largest published to date, highlighting both the reproducibility and safety of extraperitoneal RARP using the Hugo™ RAS system. Despite longer operation times and the high rate of positive surgical margins, this research work provides significant data on perioperative, functional and early oncological results associated with eRARP using this new platform.

Ongoing technological advancements, tailored training programs and system updates are crucial to overcoming initial challenges and maximizing the Hugo™ RAS system’s potential to improve surgical outcomes. To assess the non-inferiority of this new robotic platform in terms of functional and oncological outcomes, further larger prospective and randomized studies will be necessary.

## Figures and Tables

**Table 1:** Baseline patients’ characteristics.

Age, years, mean (±SD)	65.76 (±5.57)
BMI, kg/mq, median (Q1–Q3)	27 (24–29)
CCI, median (Q1–Q3)	4.5 (4–5)
ASA Score, median (Q1–Q3)	2 (2)
Previous Abdominal Surgery, *n* (%)	20 (40)
Prostate Adenomectomy, *n* (%)	4 (8)
IPSS, median (Q1–Q3)	9.5 (8–14)
QoL, median (Q1–Q3)	2 (2–3)
IIEF-5, median (Q1–Q3)	15.5 (13–19)
PI-RADS index, median (Q1–Q3)	4 (4–5)
Lesion Diameter, mm, median (Q1–Q3)	9.5 (6–14)
Preoperative PSA level, ng/mL, median (Q1–Q3)	7 (5–9.9)
Positive Digital Rectal Examination, *n* (%)	16 (32)
Prostate Volume, ml, median (Q1–Q3)	39.5 (31–47)
ISUP 1–2 at biopsy, *n* (%)	34 (68)
ISUP 3–5 at biopsy, *n* (%)	16 (32)
cN+, *n* (%)	3 (6)

SD: standard deviation; BMI: body mass index; CCI: Charlson Comorbidity Index; ASA: American Society of Anesthesiologists; IPSS: International Prostatic Symptoms Score; QoL: Quality of Life; IIEF-5: International Index of Erectile Function Questionnaire.

**Table 2:** Intraoperative data.

Pelvic lymphadenectomy, *n* (%)	26 (52)
Number of nodes removed, median (Q1–Q3)	9 (8–13)
Nerve-sparing procedure, total, *n* (%)	29 (58)
Blood loss, ml, mean (± SD)	148 (±57.4)
Intraoperative complications, *n* (%)	0 (0)
Bladder neck reconstruction, *n* (%)	2 (4)
Total surgery time (in-out), median (Q1–Q3)	315 (220–405)
- Pelvic lymphadenectomy, median (Q1–Q3)	332 (315–350)
- No pelvic lymphadenectomy, median (Q1–Q3)	300 (272.5–315)
Operative time (incision to last stich), median (Q1–Q3)	275 (150–345)
- Pelvic lymphadenectomy, median (Q1–Q3)	302.5 (270–315)
- No pelvic lymphadenectomy, median (Q1–Q3)	262.5 (237.5–277.5)
Console time, min, median (Q1–Q3)	240 (150–300)
Docking time, min, median (Q1–Q3)	10 (9–10)

SD: standard deviation; Q1–Q3: Quartiles 1 and 3.

**Table 3:** Postoperative data.

Postoperative Complication—Clavien–Dindo grade, median (Q1–Q3)	2 (1–2)
Clavien–Dindo grade I, *n*	6
- Clavien–Dindo grade II, *n*	5
- Clavien–Dindo grade III, *n*	2
- Catheter removal (POD), median (Q1–Q3)	12 (10–13)
Hb difference pre-post surgery, g/dL, median (Q1–Q3)	3.1 (2–4)
POD of discharge, median (Q1–Q3)	4 (3–5)
Narcotic use, *n* (%)	2 (4)
NSAIDS use, *n* (%)	4 (8)
Paracetamol use, *n* (%)	33 (66)
Prostate volume at final pathology, mL, median (Q1–Q3)	39.5 (35–50)
Tumor volume at final pathology, mL, median (Q1–Q3)	2.65 (1.6–4.3)
Primary Gleason at final pathology, median (Q1–Q3)	3 (3–4)
Secondary Gleason at final pathology, median (Q1–Q3)	3 (3–4)
ISUP at final pathology, median (Q1–Q3)	2 (1–3)
Perineural Invasion at final pathology, *n* (%)	32 (64)
Positive Surgical margins, *n* (%)	22 (44)
- Clinically significative positive surgical margins [18], *n* (%)	14 (63.6)
pT stage	
- pT2a, *n* (%)	4 (8)
pT2c, *n* (%)	33 (66)
- pT3a, *n* (%)	5 (10)
- pT3b, *n* (%)	8 (16)
pN stage	
- pN0, *n* (%)	23 (46)
pN1, *n* (%)	3 (6)
- pNx, *n* (%)	24 (48)
Follow-up data	
- Undetectable PSA (<0.1 ng/mL) at 1 months, *n* (%)	41 (82)
- Undetectable PSA (<0.1 ng/mL) at 3 months, *n* (%)	36 (72)
- Social continence rate at 1 months, *n* (%)	31 (62)
- Social continence rate at 3 months, *n* (%)	36 (72)
- Erectile potency at 3 months, *n* (%)	20 (40)
- IIEF-5, median (Q1–Q3)	12 (9–16)

ISUP: International Society of Urological Pathology; Q1–Q3: Quartiles 1 and 3; NSAIDS: non-steroidal anti-inflammatory drugs; POD: postoperative day; IIEF-5: International Index of Erectile Function Questionnaire.

## Data Availability

The original contributions presented in the study are included in the article, further inquiries can be directed to the corresponding author/s.
